# Three-Dimensional Printed Model-Assisted Screw Installation in Treating Posterior Atlantoaxial Internal Fixation

**DOI:** 10.1038/s41598-018-29426-2

**Published:** 2018-07-23

**Authors:** Minyi Yang, Nannan Zhang, Haodong Shi, Hui Li, Shichang Liu, Zongrang Song, Lequn Shan, Qining Wu, Dingjun Hao

**Affiliations:** 1 Honghui Hospital, Xi’an Jiaotong University College of Medicine, Xi’an, People’s Republic of China; 2 National Center for Birth Defect Monitoring, West China Second University Hospital, Sichuan University and Key Laboratory of Birth Defects and Related Diseases of Women and Children (Sichuan University), Ministry of Education, Chengdu, Sichuan 610041 China; 3 Department of Spine Surgery, Honghui Hospital, Xi’an Jiaotong University College of Medicine, Xi’an, People’s Republic of China

**Keywords:** Bone, Trauma

## Abstract

The aim of this study was to evaluate the efficacy and feasibility of a life-size 3-dimensional printing assisted posterior internal fixation. We performed a retrospective review of 138 patients who received posterior atlantoaxial internal fixation from October 2009 to March 2015 with a minimum follow-up period of 12 months. Group A included 76 patients who received the conventional free-hand technique. Group B included 62 patients who were treated with internal fixation assisted by 3D printing. The placement accuracy of the screw was evaluated in the computed tomography images according to the methods of Hojo and clinical outcomes were evaluated using the visual analogue scale, the Japanese Orthopedic Association Score, and the Neck Disability Index score. There were no significant differences in the clinical results at any of the follow-up time points regarding the JOA, VAS, or NDI scores between two group. However, compared to Group A, Group B had better results for screw installation (*P* = 0.003), shorter surgery time (*P* = 0.001), and less blood loss (*P* = 0.037). Compared to the conventional free-hand technique, 3D printed model–assisted is helpful to screw placement in atlantoaxial internal fixation, which can be used as a common tool to provides important guidance for upper cervical surgery.

## Introduction

Posterior atlantoaxial screw fixation is the standard operative treatment for atlantoaxial internal fixation^1–3^. Unlike in the transarticular screw method, a free-hand transpedicle screw may migrate into the vertebral artery (VA) foramen and lead to vertebral artery injury, which may cause cerebellar infarction or brain stem infarction. In conventional surgery, 3D-reconstructed preoperative computed tomography (CT) images can only be visualized on a computer screen or X-ray view box, and the images must be memorized for use during surgery. This approach meets the demand for surgery with higher precision and efficiency.

CT navigation can lead to the more precise placement of screws. However, the navigation system is too expensive for most hospitals; therefore, the application of this technology is limited due to factors such as a lack of equipment, insufficient training, and high costs^4–7^.

Recently, 3D printing technology has been more widely used to develop precise and personalized surgical treatments^8–10^. However, posterior atlantoaxial internal fixation has rarely been studied in the context of surgery assisted by 3D printing. This study aimed to evaluate the accuracy of screws position during posterior atlantoaxial surgery, which was assisted by 3D-printed patient-specific models.

## Materials and Methods

### Patient population

From October 2009 to March 2015, 138 patients with atlantoaxial lesions received posterior C1–C2 fixation treatment in our hospital. Indications for surgery were reducible atlantoaxial fracture and dislocation (99 cases), congenital odontoid nonunion (12 cases), atlantoaxial instability due rheumatoid arthritis (10 cases) and other causes of atlantoaxial instability (17 cases). Exclusion criteria: (1) Other internal fixation techniques. Such as lamina screw, atlas lamina hook. (2) Atlantoaxial malignant tumor. (3) Atlantoaxial pedicle is thin, and the screws cannot be fixed accurately. All patients went through static and dynamic X-ray inspection, CT scanning evaluation, and magnetic resonance imaging (MRI).

**Figure 1:**
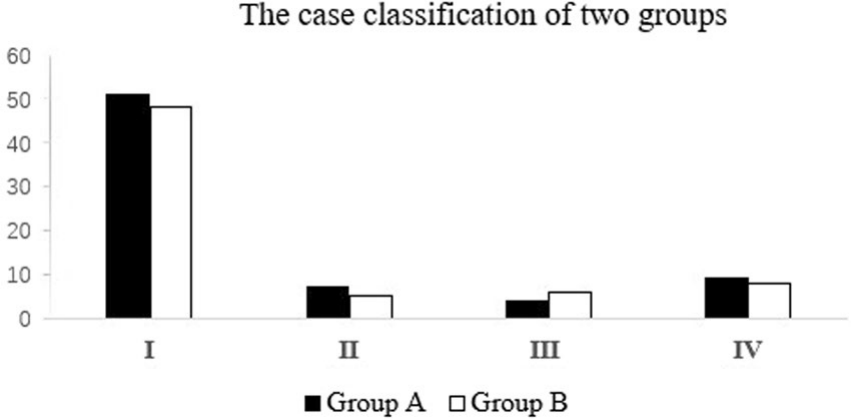
(I) Atlantoaxial fracture and dislocation. **(**II) Congenital odontoid nonunion. (III) Atlantoaxial Instability due rheumatoid arthritis. (IV) Other causes of atlantoaxial instability.

In the early stage, free-hand technique was used more frequently. 3D models were used according to doctors’ habits and patients’ wishes. In addition, some patients cannot use 3D models because they need emergency surgery. The patients were divided into 2 groups based on inclusion and exclusion criteria. Group A (n = 76) included 36 men and 40 women who treated with the traditional free-hand technique. There were 51 cases of atlantoaxial fracture accompanied dislocation, 7 cases of congenital odontoid nonunion, 4 cases of atlantoaxial instability due rheumatoid arthritis and 9 cases of atlantoaxial instability due rheumatoid arthritis. Group B (n = 62) included 26 men and 36 women treated with the assistance of 3D-printed models. There were 48 cases of atlantoaxial fracture accompanied dislocation, 5 cases of congenital odontoid nonunion, 6 cases of atlantoaxial instability due rheumatoid arthritis and 8 cases of atlantoaxial instability due rheumatoid arthritis. Group A had an average age of 51.3 years and Group B had an average age of 49.8 years. No statistically significant difference between the two groups in diagnosis and general data (Table 1, Fig. 1). All five senior spine surgeons have been involved in clinical work for more than 20 years, and each have participate in more than 1,500 spinal operations, who have similar surgical and clinical experience. The use of 3D models was dependent on surgeons’ habits and familiarity with techniques. This research was approved by the Xi’an Honghui Hospital Examining Committee and Ethics Committee. Informed consents were obtained from all participants and all methods in this study were in accordance with the Declaration of Helsinki.

**Table 1:** The case classification of two groups.

Case classification	Group A	Group B	*P* value
Atlantoaxial fracture and dislocation	51	48	0.729^△^
Congenital odontoid nonunion	7	5	
Atlantoaxial Instability due rheumatoid arthritis	4	6	
Other causes of atlantoaxial instability	9	8	
Total	76	62	

^△^*P* > 0.05 compared to the data of Group A.

### Model production by 3D printing

The 3D printing data were collected via CT scanning. The layer thickness of the CT scans was 1 mm. The C0-2 data obtained from CT scanning were saved in the DICOM format. Surgeons can use 3D printing services provided by commercial companies or 3D printers. Group B patients should go through a computed tomography angiography (CTA) examination before operations to conduct 3D printing of a VA simultaneously. DICOM data were saved as STL files through MIMICS software (Materialise Interactive Medical Image Control System Software, Materialise, Belgium), and then 1:1 models were printed using 3D printers (ProJet 360, 3D System Inc., Rock Hill, SC, USA). It took approximately 4–6 hours from the initial CT scan to obtain the models, with general costs of roughly 1000 RMB. The cost of the 3D model is included in the hospitalization.

**Figure 2:**
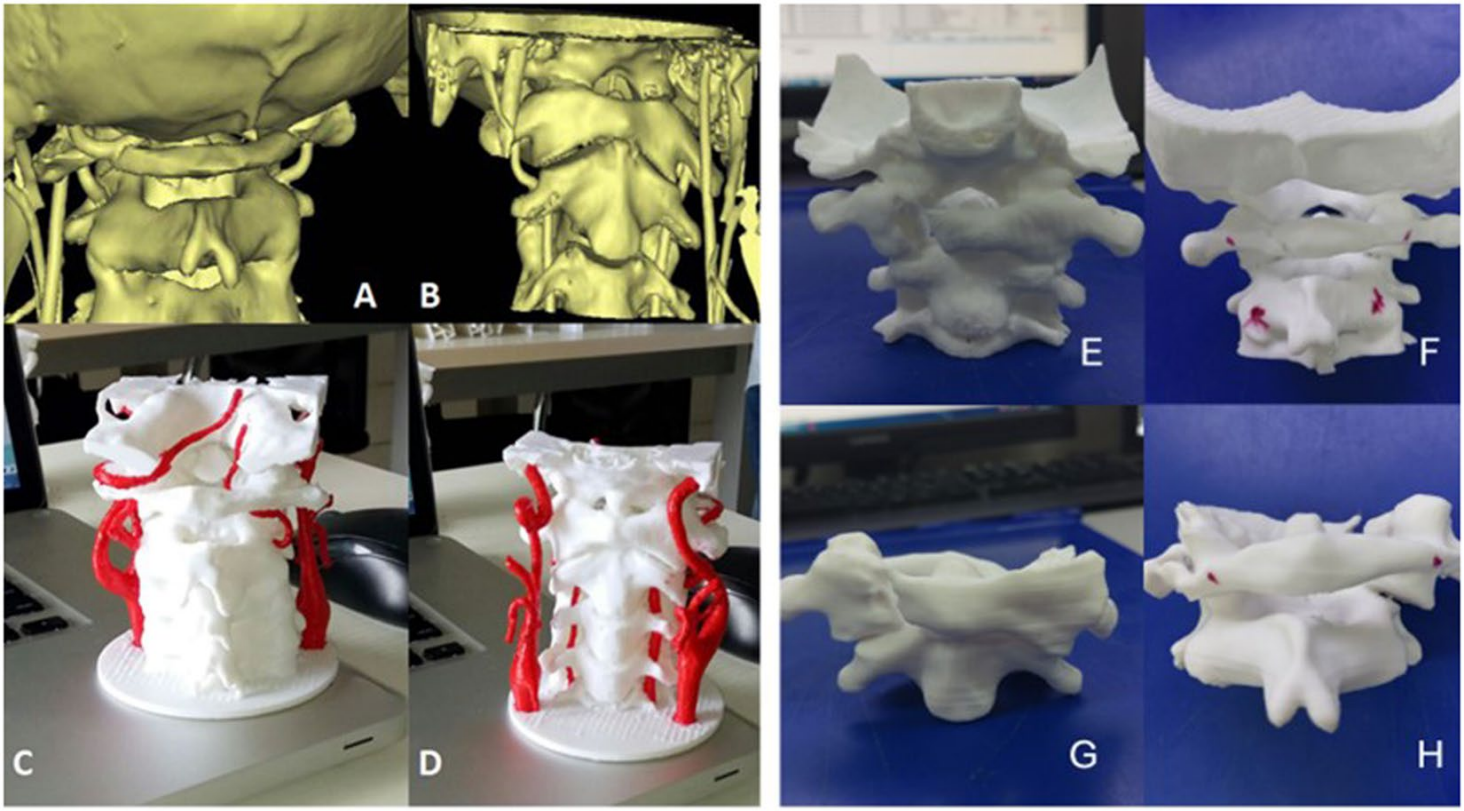
(**A**–**D**) A life-size 3D-printed C0-2 model with the vertebral artery clearly presented. (**E**,**F**) The 3D model provides more intuitive information, which can conducive to case discussion and select screw entrance point before surgery.

3D models with a scale of 1:1 were used as templates to help surgeon diagnose fracture types and make preoperative plans, select the best screw entrance point and direction. The 3D models were sterilized at low temperatures before the operation and were used during the operations to assist surgeons with identifying anatomy,which were provided more intuitive information (Fig. 2).

### Surgical technique

The screw trajectory for the C1 pedicle screw fixation was inserted using the method described by Resnick^11^. The C1 screws (diameter 3.5 mm, length 26–30 mm, Vertex, Medtronic) were inserted via the posterior arch of C1 into the lateral mass. The entry point of the C1 screw was at least 2 mm below the superior rim of the C1 posterior arch and aligned with the center of the C2 lateral mass. The trajectory was approximately 10° in the medial direction and 5° in the cephalad direction. The screw trajectory for the C2 pedicle screw placement was conducted using the method described by Harms and Melcher^12^. The entry point for the C2 screw (diameter 3.5 mm, length 26–30 mm, Vertex, Medtronic) was the cranial and medial quadrant of the isthmic surface of C2 in line with the trajectory of the pedicle. The screw trajectory was placed approximately 20° to 30° medially and in the cephalad direction along the C2 pedicle.

In the atlantoaxial fracture and dislocation group,3D model can well reflect the specific location and severity of the fracture, show the direction of dislocation. In congenital odontoid nonunion group, 3D model allow surgeons to visually understand the true shape of the odontoid and extent of atlantoaxial dislocation. In atlantoaxial instability due rheumatoid arthritis group, 3D model show the extent of atlantoaxial dislocation and the area of bone destruction. In conclusion,The 3D printed model was utilized to assist the surgeon understand the degree of atlantoaxial injuries of patients, and determine the location and direction of screw placement during the operation, particularly to reduce the rate of VA injury. In surgery, the model can be reused from multiple perspectives and provide a more 3D concept and a more realistic view for the surgeon than can 3D computed tomography pictures.

### Results evaluation and follow-up

**Figure 3:**
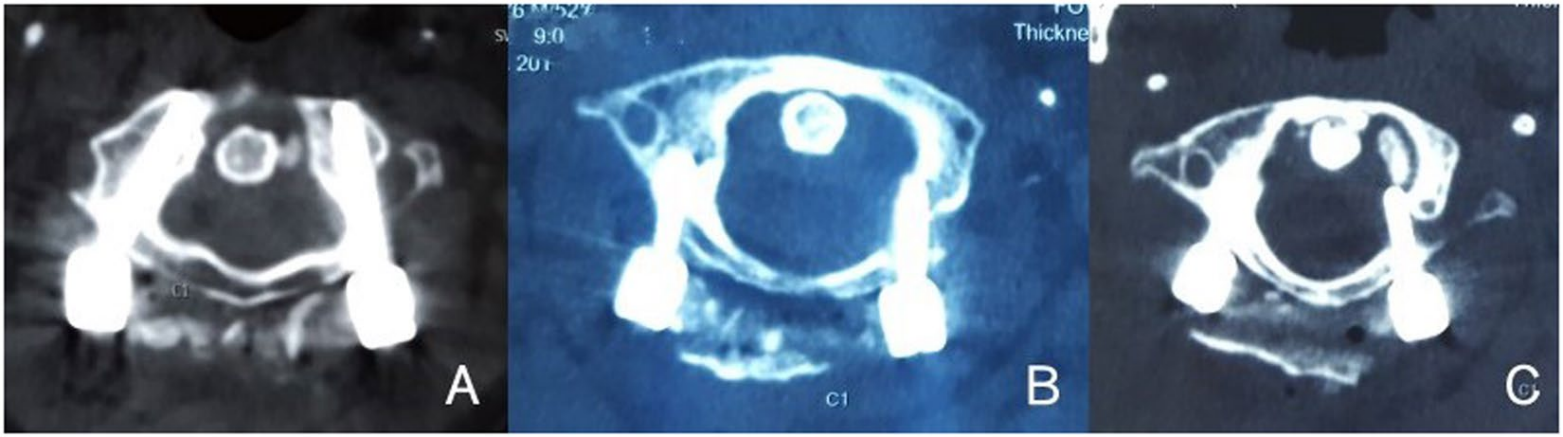
CT images were graded by the correctness of screw placement according to Hojo. (**A**) Grade 0 (G-0); (**B**) Grade 1 (G-1); (**C**) Grade 2 (G-2).

The clinical records included demographic data, operation time, blood-loss volume, hospitalization length, patient expenditures, and complications. CT images were graded by the correctness of screw placement according to Hojo^13^. Grade 0 (G0): correct position. Grade 1 (G1): position of less than half of the screw diameter was incorrect. Grade 2 (G2): position of more than half of the screw diameter was incorrect (Fig. 3).

Clinical assessments before operations and 1 week, 3 months, 6 months, 12 months after the operations were conducted according to the scoring systems of the Visual analogue scale (VAS), Japanese Orthopedic Association (JOA) and the Neck Disability Index (NDI)^14–16^. All patients with follow-up times of at least 12 months.

### Analysis of data

Statistical analyses were conducted using SPSS 17.0 software. Differences in clinical and imaging data before and after the operations were studied using paired *t* tests. Quantitative data were analyzed with *t* tests for independent samples, whereas qualitative data were analyzed with chi-square tests or rank-sum tests. *P*values less than 0.05 were considered statistically significant.

## Results

### Clinical outcomes

A total of 552 screws were placed in 138 consecutive patients during the study period. The patient distribution is summarized in Table 2. There were no significant differences between the groups in terms of age, sex, hospital costs, and hospital stay. However, Group A demonstrated greater blood loss (*P* = 0.037) and longer operation times (*P* = 0.001) than Group B.

**Table 2:** Patient demographics and surgical data.

Variable	Group A	Group B	*P* value
Age (years)	51.3 ± 7.4 (39–61)	49.8 ± 6.6 (42–59)	0.351
Males (% of group)	47.4%	41.9%	0.523
Duration of surgery (min)	159.4 ± 15.6	105.7 ± 14.6	0.001*
Blood loss(mL)	164.6 ± 28.4	114.3 ± 14.6	0.037*
Length of stay (days)	10.3 ± 2.8	9.4 ± 2.6	0.668
Follow-up (months)	42.7 (12–48)	46.1 (12–46)	0.681*
Hospital expenses(RMB)	55,489 ± 2,170.6	53,464 ± 2,308.4	0.736

^*^P < 0.05 compared to the data of Group A.

**Figure 4:**
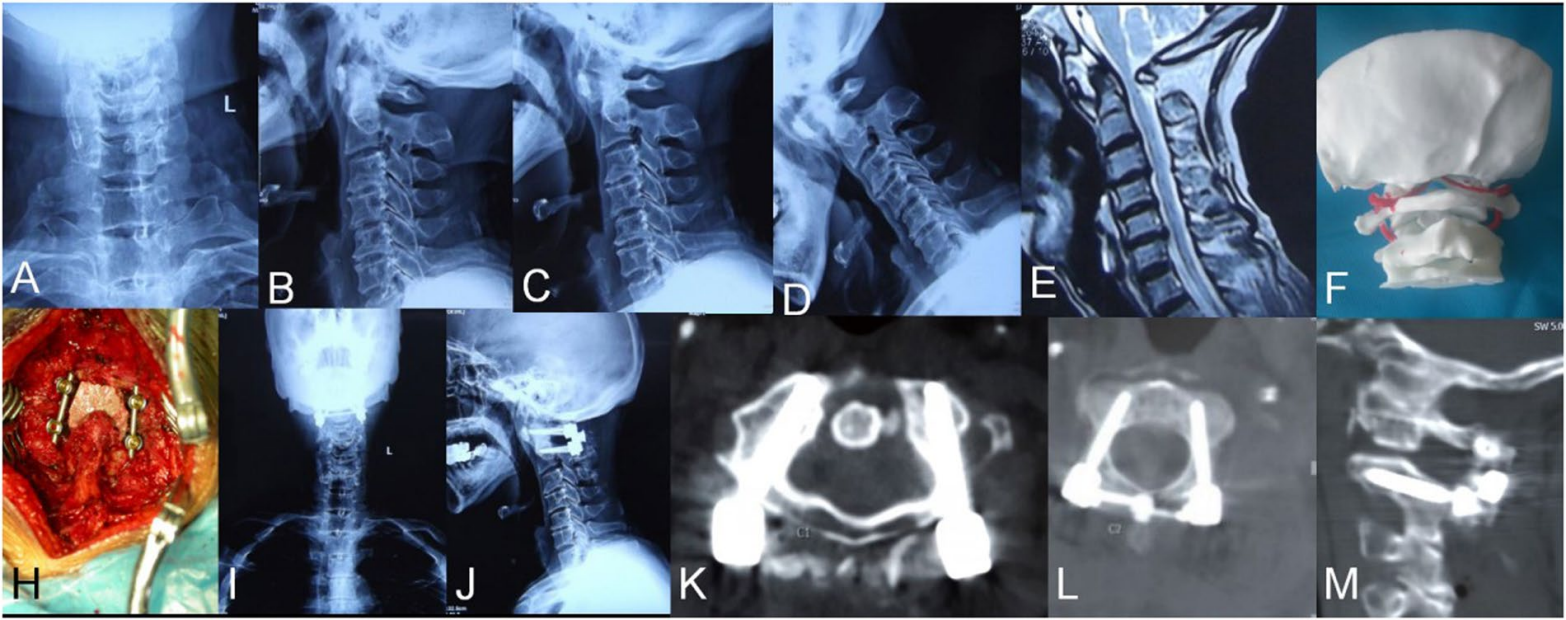
A 47 year-old male with atlantoaxial dislocation received posterior reduction and C1 pedicle-C2 pedicle internal fixation assisted by a 3D-printed life-size model. (**A**–**D**) Preoperative X-ray photographs showing atlantoaxial dislocation. (**E**) Preoperative MRI showing compression of the spinal cord. (**F**) A life-size C0-2 model. (**H**) Intraoperative photograph. (**I**–**M**) Postoperative photographs showing that the atlantoaxial dislocation was reduced and that the screw position was satisfactory.

**Figure 5:**
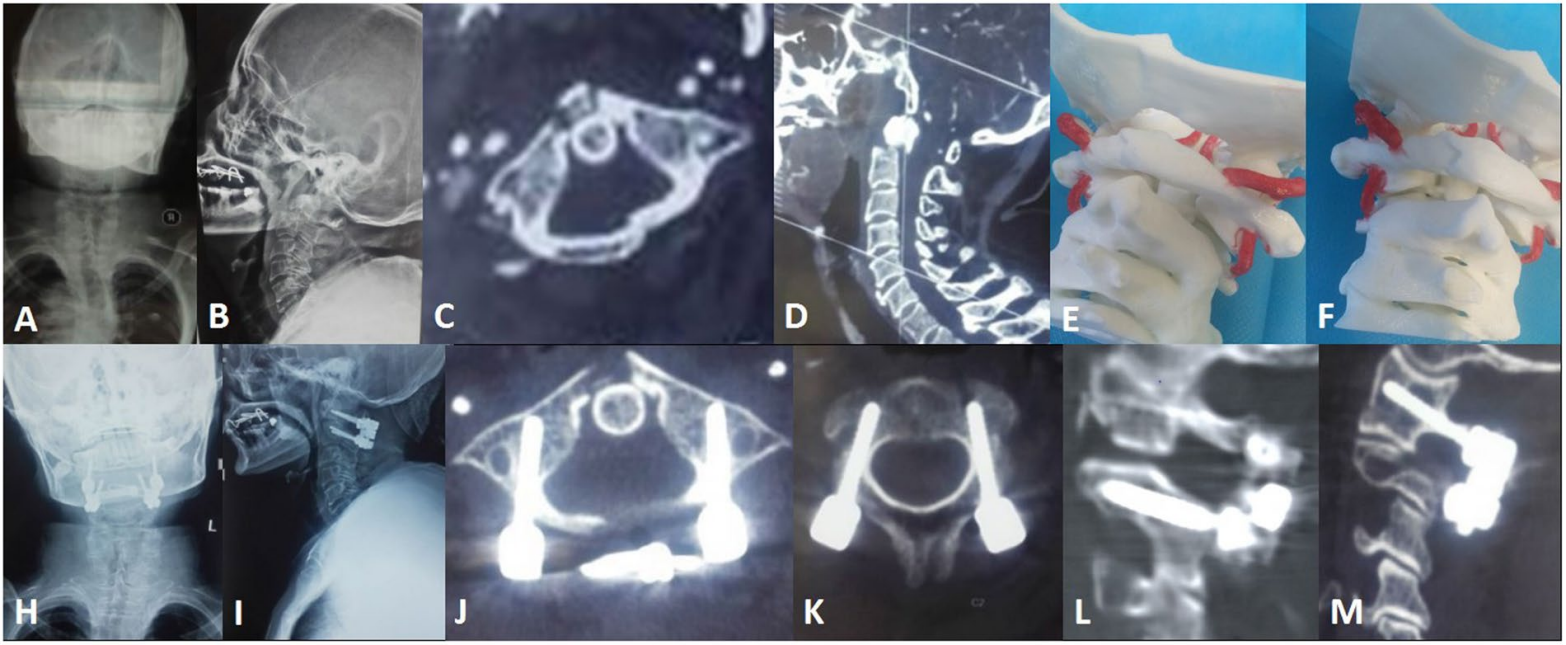
A 55 year-old female with atlantoaxial dislocation underwent posterior reduction and C1 pedicle-C2 pedicle internal fixation assisted by a 3D-printed life-size model. (**A**–**D**) Preoperative X-ray photographs showing a Jefferson fracture and odontoid fracture. (**E**) 3D life-size model showing that the vertebral artery on the left side was closer to the middle line and revealing the best screw trajectory. (**I**–**M**) Postoperative photographs showing that the screw position was satisfactory.

Detailed data are listed in Table 3. In the aspects of VAS, JOA, and NDI grading, after the operations, the patients in both groups had significant improvements in clinical indexes compared to their status before the operations, and curative effects were significant after a follow-up of 12 months. Typical cases can be seen in Figs 4, 5. However, both groups lacked significant differences (*P* > 0.05) between the temporal points at 1 week, 3 months, 6 months, and 12 months after their operations.

**Table 3:** Comparison of clinical outcomes of the two groups.

score	JOA score		VAS score		NDI score	
time	Group A	Group B	Group A	Group B	Group A	Group B
Preoperative	12.7 ± 2.1	13.1 ± 2.6	3.4 ± 0.5	3.1 ± 0.4	15.3 ± 3.2	16.4 ± 3.6
1 week	14.2 ± 1.7	13.2 ± 2.4	2.5 ± 0.3	2.1 ± 0.4	8.3 ± 1.1	8.7 ± 1.8
3 months	14.9 ± 2.5	13.8 ± 1.8	1.4 ± 0.4	2.1 ± 0.3	7.4 ± 1.9	8.1 ± 1.4
6 months	15.4 ± 2.6	15.0 ± 2.1	1.1 ± 0.8	1.4 ± 0.5	5.2 ± 1.1	4.3 ± 0.8
12 months	15.1 ± 2.0^†^	15.3 ± 2.4^†^	1.2 ± 0.7^†^	1.3 ± 0.4^†^	4.1 ± 0.6^†^	5.2 ± 1.2^†^

JOA = Japanese Orthopaedic Association; NDI = neck disability index; VAS = visual analogue scale.

^†^*P* < 0.05 compared to preoperative measurements.

### Radiographic outcomes

Two independent observers measured CT imaging data using ACDSee Canvas 11 software one week after surgery, and they were unaware to which group the patients belonged. If the grade of screw is controversial, more significant degree of breach will be used in the final analysis.

Detailed measurement data of the screw positions are listed in Tables 4, 5. Group A used 304 screws in total, of which 96 screws were rated as G1 and 2 screws were rated as G2 according to Hojo grading. Group B used 248 screws in total, of which 29 screws were rated as G1 and none screw were rated as G2 screws according to Hojo grading. The overall positions were incorrect in 32.9% in Group A and 11.3% in Group B, and significant differences occurred between two groups (Table 4). The inclusion criteria were divided into four types. Through the comparison between two groups of patients in the same type of case, the differences were statistically significant (Table 5).

**Table 4:** Comparison of the malposition of screws using CT photographs according to Hojo’s method.

Group	Grade 0	Grade 1	Grade 2	Total	*P* value
Group A	206	96	2	304	0.003^£^
Group B	219	29	0	248	
Total	425	125	2	552	

^£^*P* < 0.05 compared to the data of Group A.

**Table 5:** Comparison of the malposition of screws in case classification.

Group	Grade 0	Grade 1	Grade 2	*P* value
Group A I	162	60	2	0.000^#^
Group B I	157	19	0	
Group A II	17	11	0	0.026^#^
Group B II	18	2	0	
Group A III	7	9	0	0.003^#^
Group B III	21	3	0	
Group A IV	20	16	0	0.026^#^
Group B IV	23	5	0	

(I) Atlantoaxial fracture and dislocation (II) Congenital odontoid nonunion.

(III) Atlantoaxial Instability due rheumatoid arthritis. (IV) Other causes of atlantoaxial instability.

^#^*P* < 0.05 compared to the data of Group A.

### Complications

**Figure 6:**
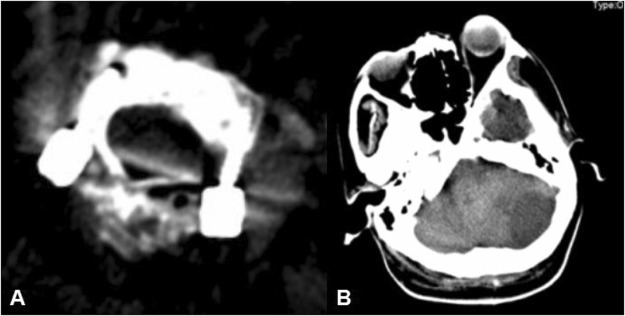
A 55 year-old female with atlantoaxial dislocation received free-hand C1 pedicle-C2 pedicle internal fixation. The bilateral vertebral arteries were involved, and the patient developed paralysis of the right upper and lower limbs approximately 4 hours postoperatively. A cerebral infarction was found in the computed tomography scan.

All patients were prepared with conventional neck supports within 8 weeks to restrict their neck movements. There were six patients in group A (G0 = 21 screws,G1 = 3 screws) and four patients in group B (G0 = 15 screws, G1 = 1 screw) had migraines, which were significantly reduced after 3 months of symptomatic treatment. A total of sixteen patients with cerebrospinal fluid leakage and no infection after the operations, including ten patients in group A (G0 = 31 screws, G1 = 9 screws), six patients in group B (G0 = 12 screws, G1 = 4 screws). One patient in Group A (G0 = 3 screws, G-2 = 1 screw) experienced cerebral infarction (Fig. 6). The myodynamia of the upper and lower limbs of the right side of that patient’s body dropped to levels 0–1. After rehabilitation exercises and trophic nerve treatment, the myodynamia of the right side of the patient’s body recovered to levels 3–4, and he could walk with support from another person. One patient in Group A (G0 = 2 screws, G1 = 1 screw, G2 = 1 screw) had quadriplegia and respiratory muscle paralysis after the operation, and he died 1 month after the operation.

## Discussion

Atlantoaxial surgery presents a considerable challenge because it has substantial risks, such as limb paralysis, VA injury, and even life-threatening emergencies. The traditional surgical approaches include the posterior approach, the anterolateral retropharyngeal approach, and the transoral approach. C1-C2 posterior screw fixation is currently the most popular technique. Despite the biomechanical superiority of cervical pedicle screws^6^, the placement of cervical pedicle screws has a considerable risk of injury, either to nerves or to the VA. Abumi *et al*.^17^ reported that 45 out of 669 inserted screws (6.7%) were misplaced in their early series. Since then, numerous reports have been published regarding the malposition rates of cervical PSs. Neo *et al*.^18^ reported that their malposition rate was 29% in patients with degenerative conditions. Onishi *et al*.^19^ reported on a patient who suffered cerebral infarction due to brain embolism after the placement of cervical PSs.

Usually the surgeon based on preoperative imaging data and intraoperative anatomic measurements to determine screw trajectory. However, The anatomic structure of the atlantoaxial pedicle has not changed as much as that of the thoracolumbar anatomical structure, and there are more factors of anatomic variation for anatomic structures of the atlantoaxial pedicle. When applying the pedicle screw technique to atlantoaxial vertebrae, individual differences should be considered^20^. For example, during the operation the small and deep operational sight makes surgeon difficult to accurately measure the patient’s anatomy with trunk bone measurement methods or image data, and individual differences among patients, some of whom suffer from posterior arch deformities. When the variation occurs in the VA, the relationship between the top of the VA loop and the pedicle may change internally and externally, which could result in serious squeezing of the pedicle by the VA and difficulty in setting the screws. Although CT can be used for 3D reconstruction before operations, it provides only a general understanding of partial anatomical structures, and the limited pictures cannot fully and truly reflect the details of many partial anatomic structures. As a result, traditional 3D images can be viewed on a 2D computer screen or exposed to actual anatomical structures during surgery, which may affect the doctor’s placement of screws in the operation.

To avoid serious complications such as VA injury, researchers attempted to improve atlantoaxial screw placement. For example, navigation systems have been used^21,22^. Intraoperative navigation has been used to help screw placement in the upper cervical spine. CT navigation can lead to more precise placement of screws. Compared with the preoperative three-dimensional CT navigation system, the navigation in the operation can reduce the difference between the data in the prone position during surgery and the preoperative CT data^23,24^. Yu *et al*.^7^ used intraoperative navigation in 23 cases (11 males, 12 females) for screw placement. The authors point out that, despite the benefits of using navigation technology, there are potential pitfalls. Wide application of navigation system is limited by a higher level of radiation (the highest dose = 40 mGy o arm two scan), cost of establishing a navigation system and daily running cost, the additional time needed for preoperative image acquisition, data transmission, and the interrupt of surgery by intraoperative navigation performance^24^. Another problem is potential navigational errors. Uehara *et al*.^25^ used the CT navigation system in 359 consecutive patients, with PS insertion of C2–L5. The perforations have a rate of 5.0% in C2 screw fixation. The author indicated that because the anatomy is highly variable, although CT-based navigation systems can result in a more precise procedure, there are still some problems at the upper cervical spine levels^26,27^.

In addition, the navigation system is too expensive for most hospitals and the application of this technology is limited due to factors such as a lack of equipment, insufficient training, and high costs. Training, technical difficulties and learning curve related issues are commonly believed to be the main barriers to acceptance of navigation surgery^28^. Furthermore, the rates of navigation system use in developing countries are lower than those in developed countries^29^. Therefore, it is necessary to develop a better method with improved accuracy and an easier operation.

At present, 3D printing technologies are commonly used in product design industries, and their use is growing in all fields, including medicine, such as orthopedic surgery, orthopedics, urology, transplant surgery, cardiothoracic surgery and pediatric surgery^30–32^. In orthopedics, 3D printing materials can be made into implants, prostheses, prosthesis production, and the creation of life-size anatomical models^33,34^. Individual 3D printing technology can contribute to surgical planning by depicting precise anatomy and thus a potential improvement in surgical outcome.

The 3D printed model provides a clear sense of anatomical structure, and 3D perspective photograph that provides much more information than 2D perspective photograph, help surgeon understand the characteristics of patients’ specific bone anatomies^35^. In the discussion and consent of complex surgical cases, 3D printed model made surgeon predicted the difficulty of the operation, assisted the preoperative plan, and selected the best surgical option^36^. In addition, the models can be sterilized by low temperature plasma and taken into the surgical region. Local anatomical structures could be intuitively revealed during the operation from multiple perspectives. During the operation, the presence of 3D model did not change that technique of screw insertion. However with individualized 3D printed model-assisted, surgeons obtain visual and tactile help, the same proportion of the anatomical mode assist surgeons select the screw insertion point, direction of screw,length of screw, and improve the accuracy of screws. To some extent, it can improve the safety of surgical procedure, reduce muscle tissue exposure and shorten operation time. The operation time and blood loss of group B were statistically significant compared with group A.

Additionally, in atlantoaxial internal fixation surgery, many patients have serious complications, such as VA infarction. However, these complications may not the result of direct damage, are caused by VA spasm due to local stimulation. A 3D printed model could facilitate the combination of screw trajectory and proper diameter and minimize the impact on the VA. The pathway of the VA and its relationship with surrounding bone structures are revealed intuitively, and thus the interference with the VA in the exposure approach at the beginning of surgery could be minimized. The use of 3D printed model may be responsible for improvement of the clinical symptoms.

With the development of 3D printing technology, production cost of the model is not expensive, it is relatively simple to make^31^. The cost of 3D printed model was 350 RMB per segment of the vertebra in our hospital. For atlantoaxial diseases, we usually choose 2–3 segments to print. So each patient will cost about 1000 RMB. Compared with the expensive cost of navigation technology, the usage fee of the navigation system is 5000 RMB per time (unrelated to the number of vertebral segments in our hospital), and the cost of 3D printed model is more acceptable to patients. The use of 3D model did not increase the economic burden of patients through statistical analysis of two groups of hospitalization expenses, further explained that when navigation is not available, 3D printed model could be used as a common tool for upper cervical surgery.

The disadvantage of this technology is that approximately 4–10 hours must be spent on obtaining CT data and producing the 3D-printed life-size model. Therefore, this approach cannot be used for patients who urgently require an operation.

The study had some limitations. First, this was a retrospective study, where patient selection existed subjective factors. In the future, prospective, randomized controlled, multicenter, large sample clinical studies will be designed. Secondly, no comparison with intraoperative navigation technology, which is research hotspot of atlantoaxial screw placement at present. More research will be done in this area in the future. In addition, no further imaging studies were conducted, such as lack of postoperative CTA examination, which can reflect the malpositioned screws and determine the exact cause of postoperative complications. Postoperative CTA should be increased in future studies.

Our data showed that the application of 3D printing technology can provide accurate data and rich information for surgeons, improve the precision of the fixation, reduce bleeding during operations, shorten surgical time, and reduce the rate of malposition complications. It can be used as a routine preoperative planning, assisted surgical simulation, act as an orientation aid during surgery. In our experience, 3D model has potential value for medical institution where cannot afford to use expensive navigation systems.

## Conclusion

Both 3D-model assisted group and free-hand technique group had significant improvements in the aspects of VAS, JOA, and NDI score before the operation. Compared to the conventional free-hand technique, application of 3D-printed model improved correctness of screw placement according to Hojo grading, reduced the operation time and blood loss. Unlike the navigation system, which is too expensive for most hospitals, a 3D-printed model could be used as a common tool to provides important guidance for upper cervical surgery.
