# Author Correction: Three-Dimensional Printed Model-Assisted Screw Installation in Treating Posterior Atlantoaxial Internal Fixation

**DOI:** 10.1038/s41598-019-44424-8

**Published:** 2019-07-02

**Authors:** Minyi Yang, Nannan Zhang, Haodong Shi, Hui Li, Shichang Liu, Zongrang Song, Lequn Shan, Qining Wu, Dingjun Hao

**Affiliations:** 1 Honghui Hospital, Xi’an Jiaotong University College of Medicine, Xi’an, People’s Republic of China; 2 National Center for Birth Defect Monitoring, West China Second University Hospital, Sichuan University and Key Laboratory of Birth Defects and Related Diseases of Women and Children (Sichuan University), Ministry of Education, Chengdu, Sichuan 610041 China; 3 Department of Spine Surgery, Honghui Hospital, Xi’an Jiaotong University College of Medicine, Xi’an, People’s Republic of China

Correction to: *Scientific Reports* 10.1038/s41598-018-29426-2, published online 23 July 2018

In the original version of this Article, Minyi Yang and Nannan Zhang were omitted as equally contributing authors. This has now been corrected in the PDF and HTML versions of the paper.

